# Supplementary material for: A Comparative Analysis on the Effect of Variety of Grape Pomace Extracts on the Ice-Templated 3D Cryogel Features
Source: Gels. 2021 Jun 23;7(3):76. doi: 10.3390/gels7030076 (PMC8293078; doi:10.3390/gels7030076)
Supplement: Supplementary file 1 [file gels-07-00076-s001.zip › gels-1261042-supplementary.pdf]

Supporting Information

# A Comparative Analysis on the Effect of Variety of Grape Pomace Extracts on the Ice-Templated 3D Cryogel Features

**Irina Elena Raschip[1*], Nicusor Fifere[1] and Maria Valentina Dinu[1]**

[1] Petru Poni Institute of Macromolecular Chemistry, Grigore Ghica Voda Alley 41A, Iasi, Romania
* Correspondence: iecoj@icmpp.ro

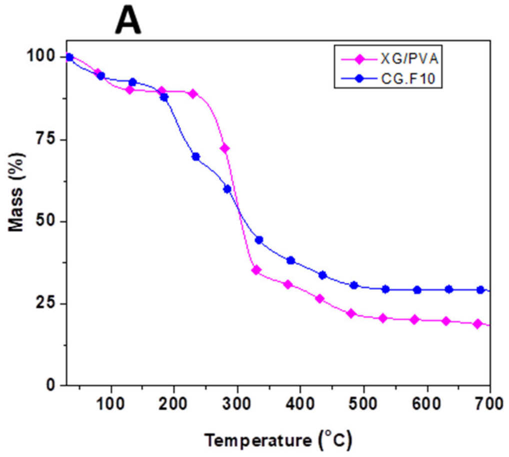


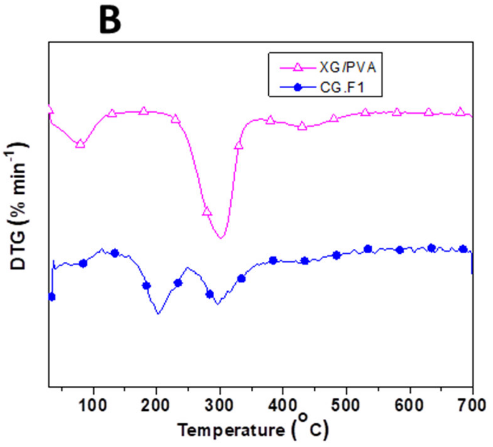


**Figure S1.** TGA (A) and DTG (B) curves of the XG/PVA and CG.F10 composite cryogels. All samples were prepared upon seven freeze-thawing cycles.
